# A comparison of three different surgery approaches and methods for neurologically intact thoracolumbar fractures: a retrospective study

**DOI:** 10.1186/s13018-021-02459-6

**Published:** 2021-05-10

**Authors:** Chao Zhu, Bin Wang, Jian Yin, Xin Hui Liu

**Affiliations:** 1 Department of Orthopedics, The Affiliated Jiangning Hospital with Nanjing Medical University, Nanjing, People’s Republic of China; 2 Department of Orthopaedic Surgery, The Affiliated Jiangning Hospital with Nanjing Medical University, No. 169 Hushan Road, Nanjing, 211100 Jiangsu Province, People’s Republic of China

**Keywords:** Pedicle screw fixation, Open, Percutaneous, Paraspinal, Thoracolumbar fracture

## Abstract

**Objectives:**

The purpose of this study was to evaluate and compare the feasibility, safety, and efficacy of conventional open pedicle screw fixation (COPSF), percutaneous pedicle screw fixation (PPSF), and paraspinal posterior open approach pedicle screw fixation (POPSF) for treating neurologically intact thoracolumbar fractures.

**Methods:**

We retrospectively reviewed 108 patients who were posteriorly stabilized without graft fusion. Among them, 36 patients underwent COPSF, 38 patients underwent PPSF, and 34 patients underwent POPSF. The clinical outcomes, relative operation indexes, and radiological findings were assessed and compared among the 3 groups.

**Results:**

All of the patients were followed up for a mean time of 20 months. The PPSF group and POPSF group had shorter operation times, lower amounts of intraoperative blood loss, and shorter postoperative hospital stays than the COPSF group (*P* < 0.05). The radiation times and hospitalization costs were highest in the PPSF group (*P* < 0.05). Every group exhibited significant improvements in the Cobb angle (CA) and the vertebral body angle (VBA) correction (all *P* < 0.05). The COPSF group and the POPSF group had better improvements than the PPSF group at 3 days postoperation and the POPSF group had the best improvements in the last follow-up (*P* < 0.05).

**Conclusion:**

Both PPSF and POPSF achieved similar effects as COPSF while also resulting in lower incidences of injury. PPSF is more advantageous in the early rehabilitation time period, compared with COPSF, but POPSF is a better option when considering the long-term effects, the costs of treatment, and the radiation times.

## Introduction

Most spinal fractures occur in the thoracolumbar segment, which is biomechanically weak against external injury [1, 2]. Although the management of thoracolumbar fractures remains controversial [3], it has been proven that surgical treatment can often achieve better clinical outcomes than conservative management [4]. Short-segment pedicle spinal instrumentation leads to the correction of kyphotic deformities, a greater initial stability, and early painless mobilization [5, 6]. However, a conventional open approach can result in some disadvantages, including blood loss, long durations of hospital stay, and injury to the paraspinal muscles [7, 8].

In 1968, Wiltse et al. first reported the paraspinal posterior open approach pedicle screw fixation (POPSF) method as a minimally invasive approach for treating lumbar spinal fractures [9]. Studies have proven that the minimally invasive approach is superior to the conventional open approach, in terms of reduced muscle injuries.

In recent years, with the rapid development of modern navigation devices, percutaneous pedicle screw fixation (PPSF) has been widely used in spinal surgery as a minimally invasive technique from the time at which it was first reported by Magerl in 1984 [10–12].

A number of clinical studies have compared PPSF to conventional open approach pedicle screw fixation (COPSF) [13–15]. Some clinical studies have also compared the paraspinal posterior open approach pedicle screw fixation (POPSF) to COPSF in the treatment of thoracolumbar fractures [16, 17]. However, few studies have compared these three approaches/methods at the same time. In this study, we compared related data from patients with neurologically intact thoracolumbar fractures who had undergone short-segment pedicle fixation, either by the conventional posterior open approach or by the minimally invasive approach containing PPSF and POPSF. The purpose of this study was to provide a scientific basis for the suitable choice of the surgical approaches for patients with thoracolumbar fractures.

## Materials and methods

### Patient samples

We retrospectively reviewed 108 cases of single-segment, neurologically intact thoracolumbar (T11-L2) fractures from January 2012 to August 2016. All of the patients were treated in the Orthopaedics Department of our hospital. The fracture type was classified as type A according to the new AO thoracolumbar fracture classification system [18]. All of the patients received operations within two weeks following the injury. The indications for operation were as follows: (a) type A1 and CA>15°, (b) dynamic fracture, and (c) kyphotic deformity getting larger after admission. These patients were divided into 3 groups based on the surgical approach and method in this study: a COPSF group (36 patients who received conventional open approach pedicle screw fixation), a PPSF group (38 patients who underwent percutaneous pedicle screw fixation), and a POPSF group (34 patients who were treated with paraspinal posterior open approach pedicle screw fixation). Before the surgery, we introduced the characteristics of three surgical approaches and methods to the patients and their family. According to patients’ conditions and permissions, we performed operations by different surgical approaches and methods. All operations were performed by the same group of doctors, and the chief surgeon was the corresponding author. All of the patients received a 4-pedicle screw fixation. We did not analyze patients who received a pedicle screw in the fractured vertebra. Procedures were performed in accordance with the Declaration of Helsinki and were approved by the Ethics Committee of Human Experimentation of our hospital. The prove reg. number was 2011030. All of the patients signed corresponding informed consents before the study. The exclusion criteria were as follows: (1) pregnancy or pathologic and osteoporotic fractures, (2) younger than 18 years old or old than 60 years old, (3) an earlier surgery had been performed at the fracture site, (4) adjacent vertebral fracture, and (5) the initial fractures were combined with other diseases that could significantly influence daily life. For patients over 50 years old, we routinely measured bone mineral density to exclude osteoporosis.

### Reduction system

In the COPSF group and POPSF group, the EXPEDIUM Spine System (DePuy Synthes, Raynham, MA, USA) was used; however, the VIPER MIS Spine System (DePuy Synthes, Raynham, MA, USA) was used in the PPSF group. All the pedicles in three groups were monoaxial. All the instrumentations in three groups were routinely removed 12 to 18 months after operation.

### Surgical procedure

#### PPSF group

**Fig. 1:**
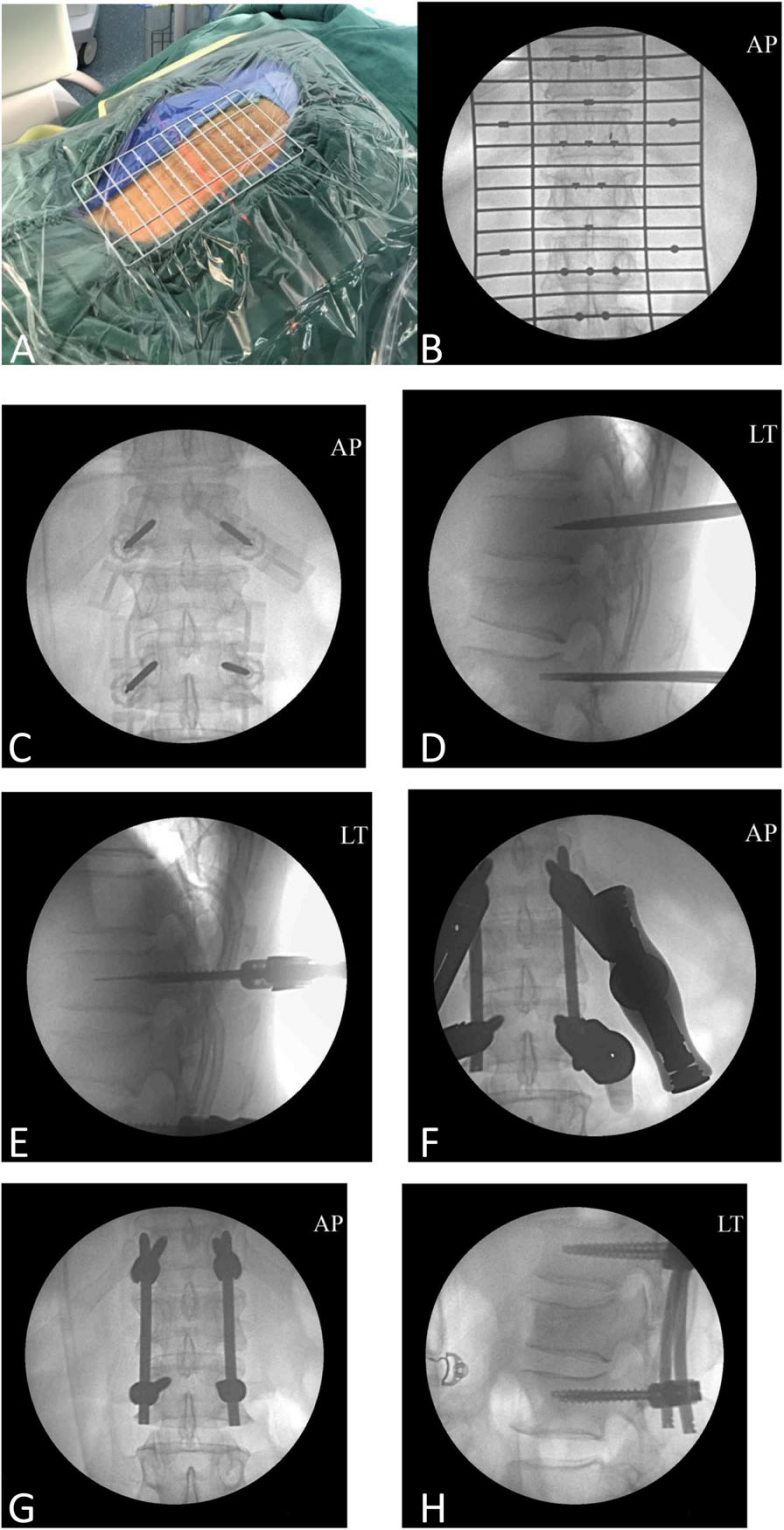
The process of the operation of percutaneous pedicle screw fixation (PPSF). **a** The preoperative locator was placed onto the back of each of the patients with the central part. **b** The correct pedicle projection and incision were obtained according to the different markers on the locator after observing the anterior-posterior fluoroscopic image. **c**, **d** The guide wire was inserted with the use of G-arm fluoroscopic image guidance. **e** The pedicle screws were inserted along the guide wire. **f** The rods were installed. **g**, **h** Postoperative radiographical images: anterior-posterior view (**g**) and lateral view (**h**)

After the general anesthesia but before the operation, the patient was place in a prone position for several minutes and the kyphosis of the injured vertebral body was partly corrected by hyperextension. We used a preoperative locator to aid in locating the pedicle projection [19]. The preoperative locator was made of stainless steel. The locator consisted of several horizontal and longitudinal rods. Different marks were made on the rods, and there were 1-cm spaces between each horizontal rod. The patient was placed into a prone position after receiving general endotracheal anesthesia, and silicone pads were used to support the chest, abdomen, and pelvis. The preoperative locator was placed on the back of each of the patients, back with the central part (Fig. 1a). The correct pedicle projection and incision were obtained according to the different markers on the locator after observing the AP fluoroscopic image (Fig. 1b). An approximately 1.5-cm incision was performed, and the underlying fascia was bluntly dissected. A puncture catheter was positioned on the outer and lower edges of the pedicle and was slowly advanced into the pedicle and posterior half of the vertebral body. The guide wire was then inserted into the catheter, and the needle was carefully removed (Fig. 1c, d). The fascia and soft tissue were separated by using a series of sequential dilators. A self-tapping, cannulated pedicle screw with an appropriate length and diameter was inserted into the vertebra through the guide wire under the protection of the outside catheter (Fig. 1e), and then, the rod was installed (Fig. 1f). The rods were fixed using screws. At first, we only tightened the screws at one end, and then lever of the screw towers of the other end to generate additional lordosis to correct the kyphosis. After the reduction, all the screws were tightened. During tapping, wire tapping, and screw implantation, the wire tapping and other instruments should be coaxial to the guide wire; otherwise, the guide wire may break through the anterior vertebral wall or pull out as the instrument enters and leaves. All of the procedures were performed with the use of G-arm fluoroscopic image guidance.

#### COPSF group

The patients in the COPSF group were treated with conventional open pedicle screw fixation surgery according to the previous study [7]. The reduction technique was the same as PPSF group.

#### POPSF group

**Fig. 2:**
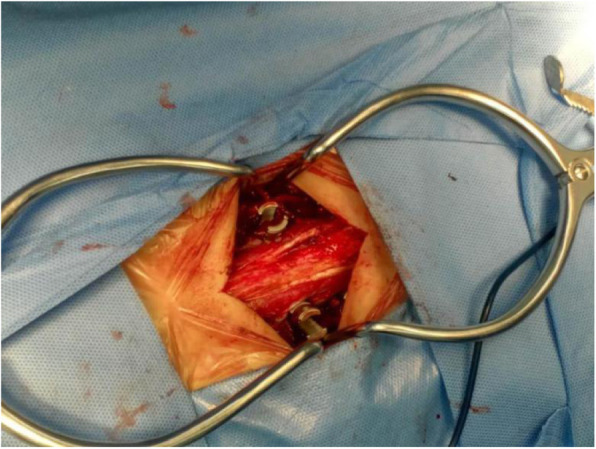
The paraspinal posterior open approach pedicle screw fixation was performed. An 8- to 10-cm posterior midline incision was performed in the target segment through the interspace between the multifidus and longissimus, in order for the pedicle entry point to be exposed

The positions of the fractured vertebrae were ascertained by using the G-arm. After routine sterilization and placement of the drapes, an approximate 8- to 10-cm midline incision was performed in the target segment. Subsequently, the thoracolumbar fascia, multifidus, and longissimus were separated in order to reach the pedicle entry point. The next procedure was the same as the traditional method (Fig. 2).

### Observation index

Three groups were compared in terms of operating time, intraoperative blood loss, intraoperative fluoroscopy, hospital stay, hospitalization cost, and postoperative complications. The vertebral body angle (VBA) and Cobb’s angle (CA) were evaluated at pre-operation, the third day after surgery, and the final follow-up. The VBA and CA were measured by conventional radiograph (X-ray). The visual analog scores (VAS) was evaluated at pre-operation, the seventh day after surgery, and the final follow-up. Oswestry disability index (ODI) scores were evaluated at pre-operation and the final follow-up. Levels of serum creatine kinase (CK) were measured at pre-operation, 1 day, and 1 week after the operation.

### Statistical analysis

All of the statistical analyses in this study were performed by using SPSS 17.0 statistical software (IL, USA). The variables with continuous data were reported as means and standard deviations. Statistical analyses were conducted by using one-way ANOVA to compare the means if they were accord with normal distribution. Where the normal distribution was not met, the Wilcoxon rank sum test was used. The categorical variables that were demonstrated as counted data were compared by using the *χ*^2^ test. *P* < 0.05 indicated statistical significance.

## Results

The demographic and clinical characteristics of a total of 108 patients, including 68 males and 40 females (mean age 43.1 years; range 24–60 years), are shown in Table 1. Injuries were due to falling from a high height in 55 patients, traffic accidents in 30 patients, and falling from a low height in 23 patients. There were 36 patients in the COPSF group, 38 patients in the PPSF group, and 34 patients in the POPSF group. In the COPSF group, 15 for type A1, 3 for type A2, 10 for type A3, and 8 for type A4. In the PPSF group, 16 for type A1, 4 for type A2, 13 for type A3, and 15 for type A4. In the POPSF group, 15 for type A1, 2 for type A2, 10 for type A3, and 7 for type A4. No patient required a posterior decompression of the vertebral canal, according to CT and MRI scans. There were no significant differences in mean age, body mass index, gender, fracture level, and fracture classification among the COPSF group, the PPSF group, and the POPSF group (all *P* > 0.05, Table 1). All of the patients were followed up for a mean time of 20 months (ranging from 14 to 38 months).

**Table 1:** Comparison of the general data among the three groups

Characteristics	COPSF group	PPSF group	POPSF group	*P* value
Cases (*n*)	36	38	34	
Mean age (years)	46.3±7.8	47.9±8.5	45.6±6.8	0.80*
Gender				
Male	21	25	22	0.78^Δ^
Female	15	13	12	
BMI	22.0±2.5	21.8±2.4	22.1±2.6	0.87*
Fracture level				
T11	6	8	4	0.93^Δ^
T12	13	13	10	
L1	11	12	13	
L2	6	6	7	
Fracture classification				
A1	15	16	15	0.94^Δ^
A2	3	4	2	
A3	10	13	10	
A4	8	5	7	

*COPSF* conventional open approach pedicle screw fixation, *PPSF* percutaneous pedicle screw fixation, *POPSF* posterior open approach pedicle screw fixation

*One-way ANOVA

^Δ^*χ*^2^ test

### Operation indexes

PPSF and POPSF resulted in reduced injuries, including shorter operation times, lower amounts of intraoperative blood loss, and shorter postoperative hospital stays, compared with the COPSF (Table 2). The PPSF group had the least amounts of intraoperative blood loss (*P* < 0.05). The intraoperative radiation times and hospitalization costs were highest in the PPSF group (*P* < 0.05), but there were no significant differences between the POPSF group and the COPSF group (*P* > 0.05). There were no significant differences among the 3 groups in terms of complications, as shown in Table 3 (*P* > 0.05).

**Table 2:** Comparison of operation indexes among the three groups

Operation indexes	COPSF group	PPSF group	POPSF group
Operating time (min)	125.3±23.4	98.4±25.3^a^	97.6±19.6^b^
Intraoperative blood loss (ml)	367.9±37.6	107.9±18.7^a^	140.1±25.8^b,c^
Intraoperative fluoroscopy (times)	3.3±1.1	28.4±1.4^a^	3.7±1.3^c^
Hospital stay (days)	14.4±1.8	10.1±2.1^a^	11.2±2.8^b^
Hospitalization cost (×10^3^ CNY)	49.9±0.9	55.2±1.0^a^	48.6±1.3^c^

One-way ANOVA was used to compare the operating times and the hospitalization costs. Wilcoxon rank sum tests were used to compare the intraoperative blood loss, intraoperative fluoroscopy, and hospital stays

*CNY* China Yuan

^a^Significant difference between the PPSF and the COPSF groups

^b^Significant difference between the POPSF and the COPSF groups

^c^Significant difference between the POPSF and the PPSF groups

**Table 3:** Comparison of the postoperative complications among the three groups

Postoperative complications	COPSF group	PPSF group	POPSF group	*P* value*
Incorrect screw positioning	1	2	2	0.82
Incision infection	1	0	0	0.36
Neurological symptom	1	2	1	0.82

*χ^2^ test

### Radiological results

Each group exhibited significant improvements in the Cobb angle (CA) and the vertebral body angle (VBA) correction (all *P* < 0.05). The COPSF group and the POPSF group had better improvements in both CA and VBA than the PPSF group at 3 days post-operation. No significant differences were observed between the COPSF group and the PPSF group in the last follow-up (*P* > 0.05), whereas the POPSF group had the best improvement in the last follow-up (*P*< 0.05) (Table 4). In all three groups, after the materials were removed, no significant changes were observed about VBA and CA. There was not anterior spondylodesis performed.

**Table 4:** Comparison of radiological parameters among the three groups

Radiological parameters	COPSF group	PPSF group	POPSF group
CA (°)			
Preoperative	17.4±5.4	17.3±5.1	18.1±5.8
3 d post	4.1±1.7	5.7±2.4^a^	4.3±2.2^c^
Last	7.6±2.8	7.9±2.7	5.9±2.9^b,c^
VBA (°)			
Preoperative	20.9±5.0	21.2±5.5	21.9±6.1
3 d post	7.0±3.0	9.1±3.2^a^	7.2±3.3^c^
Last	9.3±3.2	10.6±3.5	8.3±4.0^c^

*CA* Cobb angle, *VBA* vertebral body angle

Using one-way ANOVA

^a^Significant difference between the PPSF and the COPSF groups

^b^Significant difference between the POPSF and the COPSF groups

^c^Significant difference between the POPSF and the PPSF groups

### Effectiveness parameters

All of the groups exhibited significant reductions from baseline in the mean visual analogue scores (VAS) after the operation. There were significant differences in the VAS at 7 days after surgery among the three groups (*P* < 0.05), and it was the lowest in the PPSF group. At the last follow up, there were no significant differences in the VAS and the Oswestry disability index (ODI) score between the PPSF group and the POPSF group. However, there were significant differences in the VAS and ODI score between these two groups and the COPSF group (*P* < 0.05), and both the VAS and ODI score were the highest in the COPSF group (Table 5).

**Table 5:** Comparison of pain assessment and function evaluation among the three groups

Clinical parameters	COPSF group	PPSF group	POPSF group
VAS			
Preoperative	6.8±1.3	6.7±0.9	7.0±1.1
7 days post	3.3±1.1	2.2±0.8^a^	2.7±0.8^b,c^
Last follow-up	1.3±0.5	0.7±0.5^a^	0.8±0.6^b^
ODI			
Preoperative	90.6±3.1	90.3±2.5	90.9±2.6
Last follow-up	6.2±2.2	3.2±2.1^a^	4.1±1.9^b^

Using one-way ANOVA

*VAS* visual analogue scale, *ODI* Oswestry disability index

^a^Significant difference between the PPSF and the COPSF groups

^b^Significant difference between the POPSF and the COPSF groups

^c^Significant difference between the POPSF and the PPSF groups

### Laboratory parameters

Creatine kinase (CK) is released into the blood when skeletal muscle cells are damaged; thus, the content of serum CK levels can indicate the level of muscle damage [20]. All of the groups exhibited significant increases in the CK levels after the operation. There were significant differences in the CK levels at 1 day after surgery among the three groups (*P*<0.05). The CK levels were the highest in the COPSF group and the lowest in the PPSF group. There were no significant differences in the CK levels at 7 days after surgery among the three groups (*P* > 0.05) (Table 6).

**Table 6:** Comparison of the serum creatine kinase levels among the three groups

Laboratory parameters	COPSF group	PPSF group	POPSF group
Serum CK (U/L)			
Preoperative	215.1±68.9	223.2±55.4	205.4±50.6
1 d post	952.6±379.1	484.3±234.7^a^	690.0±232.9^b,c^
1 week post	185.5±59.6	155.4±55.7	177.5±59.1

Using one-way ANOVA

*Serum CK* serum creatine kinase

^a^Significant difference between the PPSF and the COPSF groups

^b^Significant difference between the POPSF and the COPSF groups

^c^Significant difference between the POPSF and the PPSF groups

## Discussion

The treatment of neurologically intact thoracolumbar fractures is still unclear [21, 22]. Conservative treatment has achieved satisfactory outcomes in several neurologically intact thoracolumbar fracture cases while avoiding surgical complications [23]. Most scholars believe that surgical treatment can not only correct kyphotic deformities, reduce pain, and allow patients to return to daily life activities at an earlier time but can also aid in avoiding the occurrence of delayed kyphosis and neurological symptoms [24, 25].

The neurologically intact thoracolumbar fractures were treated by the open posterior pedicle screw fixation method as previously described [7]. This approach has a clear exposure to the vertebrae and a shorter learning curve. However, it is also associated with higher infection rates, prolonged operation times, greater amounts of blood loss, and longer hospital stays [26].

Percutaneous pedicle screw fixation is classified as a minimally invasive surgery that does not require the need to peel the paraspinal muscles during the operation, thus reducing the chance of injury. Patients can recover more quickly after surgery [27]. In this study, operation times, amounts of blood loss, VAS scores, and hospital stays were significantly lower in the PPSF group than those in the COPSF group, which is consistent with previous studies [13–15].

Paraspinal posterior open approach pedicle screw fixation is also classified as a minimally invasive surgery. The blunt dissection from the interspace between the multifidus and longissimus can significantly reduce injury to the muscle [17]. In the POPSF group, operation times, amounts of blood loss, VAS scores, and hospital stays were also significantly lower than those in the COPSF group. After the muscles are damaged, creatine kinase in the muscle cells is released into the blood. Therefore, the content of serum creatine kinase levels can be used as an indicator of muscle injury [28]. Our results showed that serum creatine kinase levels were significantly lower in the PPSF group and in the POPSF group than those in the COPSF group on postoperative day 1 and were the lowest amount in this group as well. VAS and hospital stays were significantly lower in the PPSF group than those in the POPSF group. These findings suggested that although both the PPSF group and the POPSF group are classified as minimally invasive approaches, percutaneous surgery is more advantageous in early recovery than paraspinal approach surgery. Our study showed that the operation time of the PPSF group was significantly higher than that of both the COPSF group and the POPSF group, and there was no significant difference between the COPSF group and the POPSF group. Similar results were observed when comparing G-arm exposure times. The surgeons and patients received higher doses of radiation in the PPSF group. Due to the price of the implanted device, the hospitalization costs in the PPSF group were higher than those of the COPSF and POPSF groups.

It has been reported that deviations of approximately 3% in the accuracy rates of percutaneous pedicle screw insertions are unacceptable [29]. Our accuracy results were 99.3% (143/144) in the COPSF group, 98.7% (150/152) in the PPSF group, and 98.5% (134/136) in the POPSF group, and all of the complications exhibited no significant differences among the 3 groups. Although the operation of implantation of pedicle screws was not performed under direct vision, the accuracy was guaranteed, due to the use of repeated fluoroscopy during the procedure. Our results were similar to those of some other studies [13, 30].

Our radiological results indicated that the pre-operative and postoperative Cobb’s angles and VBA exhibited significant differences in all three groups. In the PPSF group, the Cobb’s angles and VBA improvements were less than those in the COPSF and POPSF groups, and no difference was found between the COPSF group and the POPSF group. Several studies have reported that, compared with mono-axial screws used in open surgery, percutaneous multiaxial screws are relatively weak in the bone-screw load, thus affecting the reduction effect [12, 14]. However, as the percutaneous minimally invasive technique reduced the injury to the paraspinal muscle, the integrity of the ligamental structures was preserved; thus, the loss of the reduction was reduced [29, 31]. There was no significant difference between the PPSF group and the COPSF group in the last follow-up, which showed that the Cobb angle correction and the VBA correction in the PPSF group were less than those in the COPSF group. The degree of paraspinal tissue injury through the use of the paraspinal posterior open approach was also lower, and the reduction loss was also lower. Our studies show that the long-term results for kyphosis correction in the POPSF group were best among the three groups.

Lee et al. [32] have shown that after lumbar muscle injury, the infiltration of adipose tissue can affect muscle contraction, thus leading to the recurrence of postoperative lower back pain (LBP). Our study showed that postoperative VAS scores were lower in the PPSF group than those in the POPSF group, but there was no difference in the long-term observation. In the final follow-up, VAS and ODI scores were highest in the open group, and we speculated that the recurrence of LBP in many patients of the COPSF was associated with muscle injury.

## Limitations

Our study has some limitations. First, it was a single-center study with small sample size. Because the numbers of patients with type A2, A3, and A4 thoracolumbar fractures were very small, we could not perform further comparative among different subtypes. Second, this study was a retrospective cohort study, so the cases were easily lost to follow-up. The last, we did not use MRI and histological and electrophysiological analyses to assess multifidus muscle damages.

## Conclusions

In conclusion, percutaneous pedicle screw fixation and paraspinal posterior open approach pedicle screw fixation are both acceptable, minimally invasive surgical-therapeutic choices for patients with neurologically intact thoracolumbar fractures. These techniques have a lot of advantages over conventional open posterior pedicle screw fixations. Percutaneous screws appear to be more advantageous in early rehabilitation time periods, but paraspinal posterior open approach pedicle screw fixation is a better option, given the long-term effects, the costs of treatment, and the amount of radiation doctors and patients receive.

## Data Availability

The data and materials contributing to this article may be made available upon request by sending an e-mail to the correspondence author.

## Abbreviations

COPSF: Conventional open pedicle screw fixation
PPSF: Percutaneous pedicle screw fixation
POPSF: Paraspinal posterior open approach pedicle screw fixation
VBA: Vertebral body angle
CA: Cobb’s angle
VAS: Visual analog scores
ODI: Oswestry disability index
CK: Creatine kinase
LBP: Lower back pain
